# Security Enhanced Anonymous Multiserver Authenticated Key Agreement Scheme Using Smart Cards and Biometrics

**DOI:** 10.1155/2014/281305

**Published:** 2014-09-08

**Authors:** Younsung Choi, Junghyun Nam, Donghoon Lee, Jiye Kim, Jaewook Jung, Dongho Won

**Affiliations:** ^1^Department of Computer Engineering, Sungkyunkwan University, 2066 Seoburo, Suwon, Gyeonggido 440-746, Republic of Korea; ^2^Department of Computer Engineering, Konkuk University, 268 Chungwondaero, Chungju, Chungcheongbukdo 380-701, Republic of Korea

## Abstract

An anonymous user authentication scheme allows a user, who wants to access a remote application server, to achieve mutual authentication and session key establishment with the server in an anonymous manner. To enhance the security of such authentication schemes, recent researches combined user's biometrics with a password. However, these authentication schemes are designed for single server environment. So when a user wants to access different application servers, the user has to register many times. To solve this problem, Chuang and Chen proposed an anonymous multiserver authenticated key agreement scheme using smart cards together with passwords and biometrics. Chuang and Chen claimed that their scheme not only supports multiple servers but also achieves various security requirements. However, we show that this scheme is vulnerable to a masquerade attack, a smart card attack, a user impersonation attack, and a DoS attack and does not achieve perfect forward secrecy. We also propose a security enhanced anonymous multiserver authenticated key agreement scheme which addresses all the weaknesses identified in Chuang and Chen's scheme.

## 1. Introduction

With the rapid growth of internet technology, a system providing various services using the network often consists of many different servers around the world. The distribution of the remote system hardware allows its users to access resources efficiently and conveniently. In multiple server environments, an authentication mechanism is required to achieve a high level of security [1]. Lamport [2] first proposed a password authentication scheme for communication through an insecure channel. However, Lamport's scheme requires the server to manage a password table and is, thus, vulnerable to stolen-verifier attacks. To resist this attack, several researchers proposed improved password-based authentication schemes using smart cards. But, these schemes are still easily broken by simple dictionary attacks due to the low entropy of passwords and because the information stored in smart cards could be extracted by physically monitoring power consumption [3, 4]. Therefore, many other researchers have combined users' biometrics and passwords to enhance the security of their user authentication schemes for multiserver environments; see, for example, references [5–7] for earlier work in this domain. Every human being has a different biometrics, and thus, it is difficult for the adversary to compute the biometric information [8, 9].

Relatively recently, D. Yang and B. Yang [10] and Yoon and Yoo [11] independently introduced a biometric-based multiserver authentication scheme. But, these schemes still do not consider user anonymity which has been identified as a major security property for privacy protection in many applications, including location-based services, anonymous web browsing, e-voting, and mobile roaming services. Moreover, D. Yang and B. Yang's scheme requires users to perform expensive exponentiation operations, while Yoon and Yoo's scheme, as demonstrated by He [12], is vulnerable to a privileged insider attack, a masquerade attack, and a stolen smart card attack.

Recently, Chuang and Chen [13] proposed an anonymous multiserver authenticated key agreement scheme to address the weaknesses in the D. Yang and B. Yang's scheme [10] and the Yoon-Yoo scheme [11]. This scheme is based on nonces and is very efficient in that it only requires users to perform hash function evaluations. Chuang and Chen claimed that their scheme satisfies all the desired security-related properties: anonymity, absence of verification tables, mutual authentication, resistance to forgery attack, resistance to modification attacks, resistance to replay attacks, fast error detection, resistance to off-line guessing attacks, resistance to insider attacks, simple and secure password choice and modification, biometric template protection, and session key agreement. However, we found that Chuang and Chen's scheme has various security problems. According to our analysis given in this paper, Chuang and Chen's scheme is vulnerable to a masquerade attack, a smart card attack, a user impersonation attack, and a denial-of-service (DoS) attack and does not achieve perfect forward secrecy. To solve these security problems with Chuang and Chen's scheme, we propose an improved anonymous multiserver authenticated key agreement scheme using a smart card together with biometrics and passwords.

The remainder of this paper is organized as follows.  Section 2 describes security and efficiency requirements for anonymous user authentication schemes in multiserver environments.  Section 3 briefly reviews Chuang and Chen's authentication scheme, while Section 4 provides a detailed security analysis on the scheme.  Section 5 presents our security-enhanced authentication scheme and shows how the security weaknesses of Chuang and Chen's scheme are addressed in our scheme.  Section 6 analyzes our scheme in terms of both security and efficiency.  Section 7 concludes the paper.

## 2. Requirements for Multiserver Authentication Schemes

**Figure 1:**
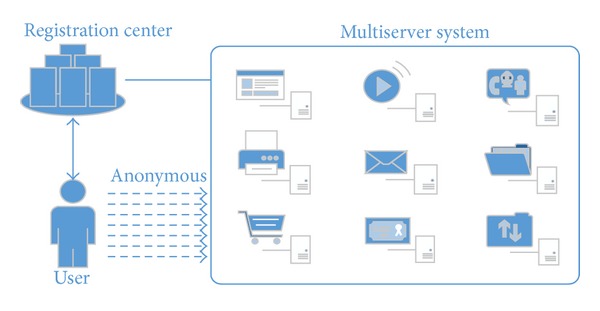
Framework of a multiserver authentication system.

Most conventional password authentication methods, when they are deployed in a multiple server environment, require each network user not only to log into various remote servers repetitively but also to remember many sets of identities and passwords. Such inefficiency and complexity easily lead to the exposure of users' identities and passwords and necessarily make it difficult to manage the shared secret keys among the involved participants. Moreover, those conventional authentication methods usually do not provide user anonymity. In contrast, an anonymous multiserver authentication scheme is designed to allow users to be authenticated by multiple servers via only one registration with the registration center [1].  Figure 1 shows a framework of an anonymous user authentication system in a multiserver environment.

### 2.1. Security Properties

Various security requirements for a multiserver authentication scheme have been suggested in the previous studies [1, 7, 10, 13–24]. The most essential security properties include the following.

(S1) *Anonymity*: anonymity is of increasing importance and is achieved when the user's identity is not disclosed to an unauthorized party.
(S2) *Mutual authentication*: mutual authentication means that the two parties, user and server, authenticate each other. That is, both user and server are assured of each other's identity.
(S3) *Session key agreement*: the user and server securely agree on a session key to be used for protecting their subsequent communications.
(S4) *Perfect forward secrecy*: perfect forward secrecy means that a session key derived from a set of long-term keys will not be compromised if one of the long-term keys is compromised in the future.

### 2.2. Attack Resistance

To achieve these security properties, a multiserver authentication scheme has to resist various kinds of attacks. The most typical attacks include the following

(A1) *Replay attack*: an adversary intercepts data transmissions for the purpose of making use of that data in some manner. Typically, this type of attack involves copying and possibly altering the data in various ways before releasing it for delivery to the intended recipient.
(A2) *Modification attack*: an adversary intercepts the authentication message and attempts to modify it for illegal authentication.
(A3) *Stolen-verifier attack*: an adversary steals the password-verifier from the server and directly uses it to masquerade as a legitimate user.
(A4) *Off-line guessing attack*: an adversary guesses a password and verifies it in an off-line environment. The information stored in the smart card is often used in such an attack.
(A5) *Forgery attack*: a malicious yet legitimate user attempts to forge an authentication message of another legitimate user.
(A6) *Insider attack*: an insider attack literally means an attack mounted by a malicious insider. Malicious insiders have a distinct advantage over external adversaries because they have an authorized system access and also may be familiar with the network architecture and system policies/procedures. Typically, malicious insiders want to acquire users' private information such as their password and biometrics.
(A7) *Masquerade attack*: an adversary is authenticated by the server using a fake user ID.
(A8) *Smart card attack*: an adversary is authenticated by the server by using only the information obtained from a user's smart card but without the password or biometrics of the user.
(A9) *User impersonation attack*: an adversary impersonates a legitimate user using only the user's smart card but without the password or biometric of the user.
(A10) *DoS attack*: a DoS attack is any event that diminishes or eliminates a network's capability of performing its expected function. In other words, an adversary mounts a DoS attack to make the server unavailable.

### 2.3. Efficiency Measures

Efficiency is an important consideration in evaluating any schemes or protocols. The efficiency of a multiserver authentication scheme can be measured by the following metrics.

(E1) * Single registration:* a single point of registration ought to allow users to gain access to all the servers in the system.
(E2) * Simple and secure password modification*: the system should allow users to choose and change their passwords easily and securely. In other words, each user should be able to change their passwords without the help of any third trusted party once the authenticity of the user is verified by its smart card.
(E3) * Fast error detection:* the smart card needs to check the user's incorrect password or any other discrepancy quickly.
(E4) * Low computational cost:* the computational cost incurred by the scheme should be minimized for the participants.

## 3. A Review of Chuang and Chen's Scheme

This section describes Chuang and Chen's anonymous multiserver authenticated key agreement scheme which involves four phases: server registration, user registration, login and authentication, and password change. For convenience, the notations used throughout this paper are summarized in Notation Section.

### 3.1. The Server Registration Phase

The application server sends the RC a join message if it would like to become an authorized server. Then, the RC replies with the key (PSK) to the server through a secure channel. And then, the authorized server uses the PSK to check the user's authentication message. If the server needs to obtain the PSK from the RC to perform the authentication phase every session, authentication delay and the communication cost between the RC and the servers will increase substantially, but this scheme and proposed scheme register only once so they are efficient.

### 3.2. The User Registration Phase

For a user user_*i*_, this phase is performed only once when user_*i*_ registers itself with the registration center RC.

1. user_*i*_ chooses his identity UID_*i*_ and password PW_*i*_ freely and inputs his biometrics BIO_*i*_ and sends the identity user_*i*_ and *h*(PW_*i*_ ⊕ BIO_*i*_) to RC via a secure channel.
2. RC computes *A*_*i*_ = *h*(UID_*i*_||*x*) and *B*_*i*_ = *h* ^2^(UID_*i*_||*x*) = *h*(*A*_*i*_) and *C*_*i*_ = *h*(PW_*i*_||BIO_*i*_) ⊕ *B*_*i*_ and *D*_*i*_ = PSK ⊕ *A*_*i*_ and issues user_*i*_ a smart card loaded with 〈UID_*i*_, *h*(), *B*_*i*_, *C*_*i*_, *D*_*i*_〉.

### 3.3. The Login and Authentication Phase

In this phase, user_*i*_ logs in to the smart card and is authenticated by  server_*j*_. In login phase, is executed to check the user's legality. The smart card can detects an error event immediately using the user's identification, password, and biometrics information. And then, the smart card computes 〈AUID_*i*_, *M*_1_, *M*_2_, *D*_*i*_〉 for the authentication. In authentication phase, the smart card sends authentication messages to the server_*j*_ after the user_*i*_ finishes the login phase successfully. The smart card never send user's real identity to execute the authentication phase for providing the user's anonymity. During the phase, the session-key establishment is conducted between user_*i*_ and server_*j*_.  Algorithm 1 depicts how the login and authentication phase works.

### 3.4. The Password Change Phase

One of the general guidelines to get better password security is to ensure that passwords are changed at regular intervals. Chuang and Chen's scheme allows legitimate users to freely change their passwords:

1. user_*i*_ inserts his smart card into a card reader and enters both the current password PW_*i*_ and the new password PW_i_*.
2. The smart card checks UID_*i*_ and *h*(PW_*i*_ ⊕ BIO_*i*_) ⊕ *C*_*i*_ = *B*_*i*_.
3. The smart card computes *C*_i_* = *C*_*i*_ ⊕ *h*(PW_*i*_ ⊕ BIO_*i*_) ⊕ *h*(PW_i_* ⊕ BIO_*i*_) and replaces *C*_*i*_ with *C*_i_*.

## 4. Security Vulnerabilities in Chuang and Chen's Scheme

We analyze Chuang and Chen's scheme and figure out some security vulnerabilities. Their scheme is vulnerable to the masquerade attack, smart card attack, user impersonation attack, and DoS attack and does not achieve perfect forward secrecy.

### 4.1. A Masquerade Attack

**Figure 2:**
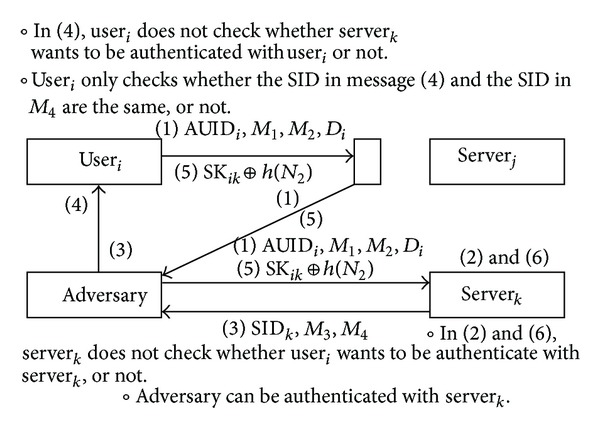
Masquerade attack on Chuang and Chen's scheme.

Chuang and Chen's scheme is vulnerable to user masquerade attack. An adversary can be authenticated to another server_*k*_ using the messages that user_*i*_ sends to server_*j*_ for authentication.  Figure 2 describes the masquerade attack on Chuang and Chen's scheme. When the user_*i*_ wants to be authenticate with server_*j*_, the user_*i*_ logs on the smart card and then sends a message (1) to the server_*j*_. After an adversary intercepts the message (1), the adversary will send it to another server server_*k*_. This is because that message (1) does not include about the server_*j*_ as follows:

(1) $$\begin{array}{c} \text{Message}\left(1\right)=\langle \text{AUI}{\text{D}}_{i},M_{1},M_{2},D_{i}\rangle ,\\ \text{AUI}{\text{D}}_{i}=h\left(N_{1}\right)⊕\text{UI}{\text{D}}_{i},\\ M_{1}=h\left(B_{i}\right)⊕N_{1},\\ M_{2}=h\left(N_{1}||\text{AUI}{\text{D}}_{i}||D_{i}\right),\\ D_{i}=A_{i}⊕\text{PSK}. \end{array}$$

So the server_*k*_ executes operation (2) and sends the message (3) to the adversary without any suspicion of the attack. The adversary forwards the message (3) to the user_*i*_. The user_*i*_ does not check the SID_*j*_ of the server_*j*_. It only checks the sameness with the SID of *M*_4_ and the SID of the message (3) as follows:

(2) $$\begin{array}{c} \text{Message}\left(3\right)=\langle \text{SI}{\text{D}}_{j},M_{3},M_{4}\rangle ,\\ M_{4}=h\left(\text{SI}{\text{D}}_{j}||N_{2}\right). \end{array}$$

So the user_*i*_ executes operation (4) and sends message (5) to server_*j*_ without any suspicion of the attack. Then, an adversary intercepts the message (5) and sends it to another server_*k*_. Finally, the adversary can be authenticated with server_*k*_. Therefore, the adversary can masquerade as a legitimate user to server_*k*_. In this way, the scheme becomes vulnerable to the masquerade attack.

The server_*k*_ cannot check whether user_*i*_ wants to be authenticated by server_*k*_ or not. Thus server_*k*_ authenticates all legitimate messages though these message are not sent to server_*k*_. And user_*i*_ does not check whether server_*j*_ wants to be authenticated with user_*i*_. Thus user_*i*_ authenticates all legitimate messages though these message are sent by server_*k*_. The user_*i*_ only checks whether SID in message (3) and SID in *M*_4_ are the same or not. To solve this problem, the destination of message is added to authentication messages. So the information about SID of server_*j*_ has to be added to the message (1), and this means that user_*i*_ want to be authenticated with server_*j*_, not server_*k*_. And the information about AUID of user_*i*_ has to be added to message (3); it means that the server_*j*_ wants to be authenticated with anonymous user_*i*_.

### 4.2. A Smart Card Attack

When an adversary gets or steals the user's smart card, the adversary can compute the session key between the user_*i*_ and server_*j*_ without the user's password or biometric information. So the adversary can decrypt the all encrypted communications between the user_*i*_ and server_*j*_ because the adversary can compute all previous session keys.  Algorithm 2 describes the smart card attack on Chuang and Chen's scheme.

When the adversary obtains the user's smart card, the adversary can extract information about the smart card using a side-channel attack such as SPA (simple power analysis) or DPA (differential power analysis). The adversary can obtain *B*_*i*_ in the user's smart card and *M*_1_, *M*_3_ in the public communication channel. Then, the adversary can compute *N*_1_ using *M*_1_ and *h*(*B*_*i*_) and *N*_2_ using *M*_3_ and *h* ^2^(*N*_1_). Finally, the adversary can determine the session key user and server using *N*_1_ and *N*_2_. This scheme uses the combination values with a password and biometrics, so the adversary cannot compute the user's password. However, using the smart card attack, the adversary can compute the session key between the user_*i*_ and the server_*j*_ without the information about user's password or biometrics.

Kocher et al. and Messerges et al. pointed out that confidential information stored in all existent smart cards could be extracted by physically monitoring power consumption [3, 4]. If a user loses his smart card, all secrets in the smart card may be revealed to the adversary. Using this information, the adversary can determine the session key between the user_*i*_ and server_*j*_. To solve this problem, it is necessary to add authentication value that adversary cannot reveal using the side-channel attack. In other words, it is necessary to add the value that only legitimate user and server can compute using the secret information, which the adversary cannot know or compute.

### 4.3. A User Impersonation Attack

**Figure 3:**
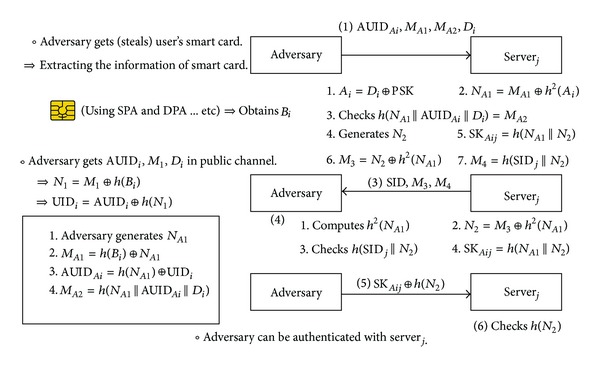
User impersonation attack on Chuang and Chen's scheme.

In Chuang and Chen's scheme, an adversary can be authenticated with the server using user's smart card without user's password or biometrics, so the adversary can impersonate the legitimate user. It is critical problem that the adversary can be authenticated with the server using user's smart card only.  Figure 3 describes the user impersonation attack on Chuang and Chen's scheme. As described above, the adversary can illegally extract the secret values including *B*_*i*_ from the user's smart card by some means. And he can intercept the message (1) = 〈AUID_*i*_, *M*_1_, *M*_2_, *D*_*i*_〉 and acquire the AUID_*i*_, *M*_1_, and *D*_*i*_.

Next procedure for user impersonation attack occurs in the following steps. The adversary computes the *N*_1_ using *M*_1_ and *h*(*B*_*i*_). And then, he can figure out the UID_*i*_ using AUID_*i*_ and *h*(*N*_1_). Next, the adversary generates another random nonce *N*_*A*1_ and computes *M*_*A*1_, AUID_*Ai*_, and *M*_*A*2_. Next, the adversary sends AUID_*Ai*_, *M*_*A*1_, *M*_*A*2_, and *D*_*i*_ to server_*j*_. The adversary can be authenticate to server_*j*_ because he knows *B*_*i*_, *N*_*A*1_, and UID_*i*_ and the server_*j*_ cannot figure out the difference between the adversary and legitimate user. The user's password and biometric information are not used in authentication phase, so server_*j*_ authenticates the adversary without doubt. server_*j*_ does not store user's password or biometric information because Chuang and Chen's scheme is designed for anonymous user. Therefore, server cannot check the password or biometric information for authentication. To solve this problem, it is necessary to add the shared value between the user and servers. The share value can be computed by only the legitimate user using user's password and biometircs in login and authentication phase, and never be stored in the smart card.

### 4.4. A DoS Attack

**Figure 4:**
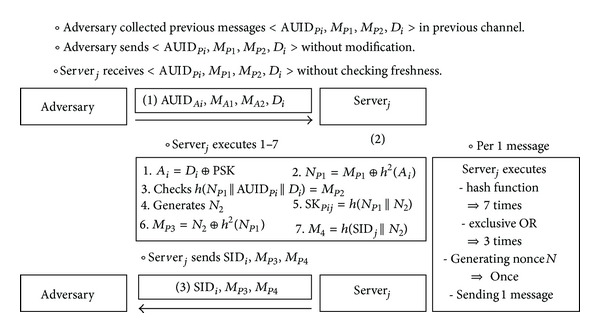
DoS attack on Chuang and Chen's scheme.

The DoS attack is an attempt to make a machine or network resource unavailable to its intended users. Although the means to carry out motives for and targets of the DoS attack may vary, it generally consists of efforts to temporarily or indefinitely interrupt or suspend services of a host connected to the networks. In Chuang and Chen's scheme, an adversary can implement the DoS attack without difficulty.  Figure 4 describes DoS attack on Chuang and Chen's scheme. The adversary gets the previous message (1) from a legitimate user and sends it to the server_*j*_. Then, the server_*j*_ executes operation (2) and sends message (3) to the user_*i*_. The processes of operation (2) include executing the hash function 7 times, calculating the exclusive-or operation 3 times, and generating a random nonce once. The adversary can attempt to make the server or network resource unavailable if he uses a lot of intercepted authentication messages.

In Chuang and Chen's scheme, server_*j*_ does not check the freshness of authentication message from user_*i*_. Thus, when an adversary sends the intercepted authentication messages to server_*j*_, the server_*j*_ cannot know whether the message is current or outdated. So, server_*j*_ executes a lot of operations. To resist the DoS attack, the server_*j*_ has to check the freshness of messages using the timestamp or other means.

### 4.5. No Perfect Forward Secrecy

Perfect forward secrecy means that a session key derived from a set of long-term keys will not be compromised if one of the long-term keys is compromised in the future. Chuang and Chen's scheme does not achieve perfect forward secrecy. So the adversary can compute the all session key between the user_*i*_ and server_*j*_ if the adversary knows the one of long-term keys *A*_*i*_ in future.  Algorithm 3 describes why Chuang and Chen's scheme does not achieve perfect forward secrecy. First, the adversary got *M*_*P*1_ and *M*_*P*3_ in previous communication between user_*i*_ and server_*j*_. Next, the adversary knows one of user's long-term secrets *A*_*i*_. So the adversary can calculate *N*_*P*1_ from *N*_*P*1_ = *M*_*P*1_ ⊕ *h* ^2^(*A*_*i*_) and *N*_*P*2_ from *N*_*P*2_ = *M*_*P*3_ ⊕ *h* ^2^(*N*_*P*1_). Finally, the adversary can compute the previous session key SK_*Pij*_ using *N*_*P*1_ and *N*_*P*2_ Therefore, this scheme does not achieve perfect forward secrecy.

In Chuang and Chen's scheme, *A*_*i*_ is a secure shared key among RC and authenticated user_*i*_. The RC computes *A*_*i*_ using UID_*i*_ and secret value *x*. And then, The RC sends the *h*(*A*_*i*_) to user_*i*_ within user's smart card. The *h*(*A*_*i*_) is unchanged even if user_*i*_ changes his password. So *A*_*i*_ is one of the long-term keys. If an adversary got the *M*_*P*1_ and *M*_*P*3_ in previous public channel and knows *A*_*i*_ at present, the adversary can compute the previous session key between the user_*i*_ and server_*j*_. To solve this problem, it is needed that the adversary cannot compute the *N*_1_ and *N*_2_ using only *A*_*i*_. By adding another secret information, it is necessary that the adversary cannot compromise the session key between user_*i*_ and server_*j*_.

## 5. Our Proposed Scheme

Our proposed scheme improves Chuang and Chen's scheme in various aspects: (1) it checks the destination of messages and so it prevents the masquerade attack, (2) it withstands the smart card attack and the user impersonation attack even when the information in the smart card is disclosed, (3) it resists DoS attacks by checking the freshness of messages, and (4) it protects the security of previously-established session keys even when the adversary knows the long-term key *A*_*i*_, thereby achieving perfect forward secrecy.

### 5.1. Countermeasures

The vulnerability of Chuang and Chen's scheme to the masquerade attack is due to the fact that

- there is no way for server_*j*_ to check whether the user wants to be authenticated with it or with another server, server_*k*_;
- user_*i*_ cannot check whether the server wants to be authenticated with him or with another user, user_*j*_.

This design flaw allows the adversary to be authenticated with server_*k*_ using user_*i*_'s message directed to server_*j*_. Therefore, to prevent the masquerade attack, we suggest to modify the computations of *M*_2_ and *M*_4_ from *M*_2_ = *h*(*N*_1_||AUID_*i*_||*D*_*i*_) and *M*_4_ = *h*(SID_*j*_||*N*_2_) to

(3) $$\begin{array}{c} M_{2}=h\left(N_{1}||\text{AUI}{\text{D}}_{i}||D_{i}||\text{SI}{\text{D}}_{j}\right),\\ M_{4}=h\left(\text{SI}{\text{D}}_{j}||N_{2}||\text{AUI}{\text{D}}_{i}\right). \end{array}$$

The server ID, SID_*j*_, and the anonymous user ID, AUID_*i*_, are now included as part of the inputs of the hash function. The inclusion of SID_*j*_ and AUID_*i*_ allows server_*j*_ and user_*i*_ to confirm the destination of the messages *M*_2_ and *M*_4_, respectively, and therefore effectively prevents the masquerade attack.

The Dos attack is possible because server_*j*_ performs all its operations without checking the freshness of incoming messages, and thus it can be prevented by modifying the computation of *M*_2_ to

(4) $$\begin{array}{c} M_{2}=h\left(N_{1}||\text{AUI}{\text{D}}_{i}||D_{i}||\text{SI}{\text{D}}_{j}||T_{i}\right), \end{array}$$

where *T*_*i*_ is the timestamp retrieved by user_*i*_ and sent to server_*j*_. The inclusion of the timestamp *T*_*i*_ to the computation of *M*_2_ enables server_*j*_ to check and confirm the freshness of the user's authentication message and prevents the DoS attack. Due to this modification, the authentication message of user_*i*_ should be also modified as follows:

(5) $$\begin{array}{c} \langle \text{AUI}{\text{D}}_{i},M_{1},M_{2},D_{i}\rangle ⟶\langle \text{AUI}{\text{D}}_{i},M_{1},M_{2},D_{i},T_{i}\rangle . \end{array}$$

We next present a possible way of eliminating the vulnerability of Chuang and Chen's scheme to the smart card attack. Recall that this vulnerability is due to that the value *B*_*i*_ stored in the smart card together with *M*_1_ and *M*_3_ exchanged between user_*i*_ and server_*j*_ enables the adversary to compute *N*_1_ and *N*_2_ and thereby to derive the session key SK_*ij*_ = *h* ^2^(*N*_1_||*N*_2_). Therefore, to prevent the smart card attack, we suggest to modify the computations of *M*_1_ and *M*_3_ from *M*_1_ = *h*(*B*_*i*_) ⊕ *N*_1_ and *M*_3_ = *N*_2_ ⊕ *h* ^2^(*N*_1_) to

(6) $$\begin{array}{c} M_{1}=h\left(B_{i}\right)⊕N_{1}⊕h\left(\text{PSK}\right),\\ M_{3}=N_{2}⊕h^{2}\left(N_{1}\right)⊕h\left(\text{PSK}\right). \end{array}$$

With this modification, the adversary now cannot compute *N*_1_ and *N*_2_ without the hash value *h*(PSK). To make this countermeasure work, we add a new value *E*_*i*_ = *h*(PSK) ⊕ *h*(PW_*i*_ ⊕ BIO_*i*_) to user_*i*_'s smart card so that only user_*i*_ can extract *h*(PSK) from its password and biometrics.

However, with the modifications described above, Chuang and Chen's scheme is still vulnerable to the user impersonation attack as the adversary can obtain *h*(PW_*i*_ ⊕ BIO_*i*_) from *B*_*i*_ and *C*_*i*_ = *h*(PW_*i*_ ⊕ BIO_*i*_) ⊕ *B*_*i*_ which are stored in the smart card. To prevent the user impersonation attack, we modify the computation of *C*_*i*_ to

(7) $$\begin{array}{c} C_{i}=h\left(\text{P}{\text{W}}_{i}⊕\text{BI}{\text{O}}_{i}\right)⊕B_{i}⊕h\left(\text{PSK}\right). \end{array}$$

The adversary now cannot calculate *h*(PW_*i*_ ⊕ BIO_*i*_) as it does not know *h*(PSK).

Finally, to provide the perfect forward secrecy in our proposed scheme, we modify the computation of *D*_*i*_ from *D*_*i*_ = PSK ⊕ *A*_*i*_ to

(8) $$\begin{array}{c} D_{i}=\text{PSK}⊕A_{i}⊕h\left(\text{PSK}\right). \end{array}$$

With this modification, the adversary cannot derive PSK from the long-term key *A*_*i*_ and, thus, cannot compute *N*_1_, *N*_2_, and the previous session key SK_*ij*_ = *h*(*N*_1_||*N*_2_).

The password update phase should be also modified for consistency purpose (see Section 5.5 for details). Combining all the modifications above together yields an improved authentication scheme described in the following subsections.

### 5.2. The Server Registration Phase

The application server sends a message for join to the RC when they want to become an authorized server. Then, the RC sends the key(PSK) to the server using secure communication. And then, the server is ready to compute *h*(PSK) for user authentication. Next, the authorized server uses the shared information like PSK and *h*(PSK) to check the user's legitimacy in authentication phase.

### 5.3. The User Registration Phase

The registration phase of proposed scheme is described in Algorithm 4. user_*i*_ needs to perform the user registration phase with the registration center using a secure channel. In this phase, RC sends to user_*i*_ the information about PSK and *h*(PSK). PSK is included in *D*_*i*_ = PSK ⊕ *A*_*i*_ ⊕ *h*(PSK). user_*i*_ can be authenticated with server_*j*_ using *D*_*i*_ but cannot compute the PSK and *A*_*i*_ even if he knows the *D*_*i*_ and *h*(PSK). And user_*i*_ can calculate the *h*(PSK) using user's password and biometrics from *E*_*i*_ = *h*(PSK) ⊕ *h*(PW_*i*_ ⊕ BIO_*i*_). In other words, the user_*i*_ receives the hidden PSK and *h*(PSK) in *D*_*i*_ and *E*_*i*_, respectively, included in smart card for user's login and authentication. Detailed steps are explained as follows.

(1) The user_*i*_ sends UID_*i*_ and *h*(PW_*i*_ ⊕ BIO_*i*_) to the RC through a secure channel.
(2) After receiving the user_*i*_'s information, the RC computes the authentication parameters for the user_*i*_ as follows:
  (9) $$\begin{array}{c} A_{i}=h\left(\text{UI}{\text{D}}_{i}||x\right),\\ B_{i}=h^{2}\left(\text{UI}{\text{D}}_{i}||x\right)=h\left(A_{i}\right),\\ C_{i}=h\left({\text{PW}}_{i}⊕\text{BI}{\text{O}}_{i}\right)⊕B_{i},\\ D_{i}=\text{PSK}⊕A_{i}⊕h\left(\text{PSK}\right),\\ E_{i}=h\left(\text{PSK}\right)⊕h\left(\text{P}{\text{W}}_{i}⊕\text{BI}{\text{O}}_{i}\right). \end{array}$$
(3) The RC stores these authentication parameters 〈UID_*i*_, *h*(), *B*_*i*_, *C*_*i*_, *D*_*i*_, *E*_*i*_〉 in a smart card and sends the smart card to user_*i*_ via a secure channel.

The RC does not store the user's password or biometrics information. Therefore, our proposed scheme is secure against a stolen-verifier attack. The registered user cannot fake another legitimate user successfully though the user obtains these parameters 〈UID_*i*_, *h*(), *B*_*i*_, *C*_*i*_, *D*_*i*_, *E*_*i*_〉. This is because that the user does not know the secret value *x* and PSK. The authenticated user can only compute *h*(PSK) using his password and biometrics.

### 5.4. The Login and Authentication Phases

The login and authentication phases for the proposed scheme are described in Algorithm 5. In the login phase, the smart card checks the legitimacy of the user. The smart card checks an error event immediately using identification, password, and biometric information. Detailed steps of the login phase are explained as follows.

(1) The user_*i*_ inserts his smart card into a card reader and enters his UID_*i*_ and PW_*i*_. Then, the user_*i*_ inputs his biometric information BIO_*i*_ using the sensor.
(2) The smart card checks the UID_*i*_ and confirms that *B*_*i*_ in smart card is same to *h*(PW_*i*_ ⊕ BIO_*i*_) ⊕ *C*_*i*_. If all information is accurate, then the smart card generates a random nonce *N*_1_ and a timestamp *T*_i_ and computes the *h*(PSK) using *E*_*i*_ and *h*(PW_*i*_ ⊕ BIO_*i*_). Next the smart card computes the following:
  (10) $$\begin{array}{c} M_{1}=h\left(B_{i}\right)⊕N_{1}⊕h\left(\text{PSK}\right),\\ \text{AUI}{\text{D}}_{i}=h\left(N_{1}\right)⊕\text{UI}{\text{D}}_{i},\\ M_{2}=h\left(N_{1}||\text{AUI}{\text{D}}_{i}||D_{i}||\text{SI}{\text{D}}_{j}||T_{i}\right). \end{array}$$

In the authentication phase, the smart card sends an authentication message to the server after the user_*i*_ finishes the login phase successfully. The proposed scheme only uses the anonymous identity AUID_*i*_ to perform the authentication phase. The detailed steps of the authentication phase are explained as follows.

(3) The smart card sends the message 〈AUID_*i*_, *M*_1_, *M*_2_, *D*_*i*_, *T*_*i*_〉 to the server_*j*_ for the user_*i*_'s authentication.
(4) The server_*j*_ confirms the legality of the user_*i*_ and the freshness of authentication message. First, the server_*j*_ checks the freshness of *T*_*i*_. If *T*_*i*_ is not fresh, the server_*j*_ rejects the user_*i*_'s request. The server_*j*_ uses PSK and *h*(PSK) to obtain *A*_*i*_ from the *D*_*i*_. The server_*j*_ computes the value of *N*_1_ (*N*_1_ = *M*_1_ ⊕ *h* ^2^(*A*_*i*_) ⊕ *h*(PSK)) and then confirms whether *h*(*N*_1_||AUID_*i*_||*D*_*i*_||SID_*j*_||*T*_*i*_) is same to *M*_2_. If the result of *M*_2_ is not same, the server_*j*_ terminates this session. Then, the server_*j*_ computes UID_*i*_ using* h*(*N*_1_) and checks the legitimacy of UID_*i*_. Next, the server_*j*_ generates a random nonce *N*_2_ and computes the following:
  (11) $$\begin{array}{c} \text{S}{\text{K}}_{ij}=h\left(N_{1}||N_{2}\right),\\ M_{3}=N_{2}⊕h^{2}\left(N_{1}\right)⊕h\left(\text{PSK}\right),\\ M_{4}=h\left(\text{SI}{\text{D}}_{j}||N_{2}||\text{AUI}{\text{D}}_{i}\right). \end{array}$$
(5) The server_*j*_ sends back the authentication message 〈SID_*j*_, *M*_3_, *M*_4_〉 to the smart card.
(6) The smart card confirms the legality of the server_*j*_. It computes *h* ^2^(*N*_1_) and then calculates *N*_2_ using *M*_3_, *h* ^2^(*N*_1_), and *h*(PSK). Next, the smart card checks whether
  (12) $$\begin{array}{c} h\left(\text{SI}{\text{D}}_{j}||N_{2}||\text{AUI}{\text{D}}_{i}\right)=M_{4}. \end{array}$$
  Next, the smart card computes the session key SK_*ij*_ as *h*(*N*_1_||*N*_2_). Finally, the smart card computes SK_*ij*_ ⊕ *h*(*N*_2_).
(7) The smart card sends the message 〈SK_*ij*_ ⊕ *h*(*N*_2_)〉 to the server_*j*_.
(8) The server_*j*_ uses the session key SK_*ij*_ for checking SK_*ij*_ ⊕ *h*(*N*_2_), anf if *h*(*N*_2_) is correct, the server_*j*_ authenticates the user_*i*_. From now on, the server_*j*_ can communicate securely with user_*i*_ using the SK_*ij*_

### 5.5. The Password Change Phase

The password change phase for the proposed scheme is described in Algorithm 6. The proposed password change phase is executed when the user_*i*_ wants to update his password. In this phase, the user_*i*_ can easily change his password without any assistance from the registration center. Detailed processes are as follows.

(1) The user_*i*_ inserts his smart card into a card reader and enters both the current password PW_*i*_ and the new password PW_i_* with UID_*i*_ and BIO_*i*_.
(2) The smart card checks UID_*i*_ and computes *h*(PSK) = *E*_*i*_ ⊕ *h*(PW_*i*_ ⊕ BIO_*i*_) and then checks whether
  (13) $$\begin{array}{c} h\left(\text{P}{\text{W}}_{i}⊕\text{BI}{\text{O}}_{i}\right)⊕C_{i}⊕h\left(\text{PSK}\right)=B_{i}. \end{array}$$
(3) The smart card computes *C*_i_* = *C*_*i*_ ⊕ *h*(PW_*i*_ ⊕ BIO_*i*_) ⊕ *h*(PW_i_* ⊕ BIO_*i*_) and then replaces *C*_*i*_ with *C*_i_*.

## 6. Analysis of Our Scheme

An anonymous multiserver authenticated key agreement scheme has three important requirements: the security properties, the attack resistance, and the efficiency, so it needs to analyze the proposed scheme using them. In this section, we explain how the proposed scheme is satisfied with the requirements and compare the proposed scheme with other authentication schemes.

### 6.1. Security Properties

(S1) *Anonymity*: in the proposed scheme, an adversary cannot compute the user's real identity UID_*i*_ without *h*(*N*_1_) because the real identity of user is always converted using AUID_*i*_ = *h*(*N*_1_) ⊕ UID_*i*_. Only legitimate server can compute and check the user's real identity, because the server has the PSK and can compute the *N*_1_ from *N*_1_ = *M*_1_ ⊕ *h* ^2^(*A*_*i*_) ⊕ *h*(PSK) using the PSK, *M*_1_, and *A*_*i*_. Thus, only authorized server confirms the UID of user. As a result, the adversary cannot obtain the user's real identity, but legitimate user_*i*_ can anonymously be authenticated with server_*j*_.
(S2) *Mutual authentication*: the mutual authentication means that two parties authenticate each other. In proposed scheme, the user and server authenticated each other using *N*_1_, *N*_2_, *h*(PSK), and *D*_*i*_. In the authentication phase, the server authenticates the user if the *M*_2_ is correct as follows:
  (14) $$\begin{array}{c} M_{2}=h\left(N_{1}||\text{AUI}{\text{D}}_{i}||D_{i}||\text{SI}{\text{D}}_{j}||T_{i}\right). \end{array}$$
  And the user authenticates the server using *M*_4_ and *N*_2_; it checks whetherthe *M*_4_ is correct as follows:
  (15) $$\begin{array}{c} M_{4}=h\left(\text{SI}{\text{D}}_{j}||N_{2}||\text{AUI}{\text{D}}_{i}\right). \end{array}$$
  Though an adversary intercepts the messages and wants to fake a legitimate user/server, the adversary cannot compute the accurate values, so it cannot send valid reply message to the user/server. This is because that the adversary does not know the secret key PSK, *h*(PSK) and random nonce *N*_1_ and *N*_2_.
(S3) *Session key agreement*: in the proposed scheme, the user and server can share the session key after the authentication phase. Then, they can communicate securely using the shared session key, which encrypts the communication packets. The session key is generated using *h*(*N*_1_||*N*_2_). *N*_1_ and *N*_2_ change in every session, so session key is different in each session. Therefore, it is difficult for the adversary to compute the session key from the intercepted messages.
(S4) *Perfect forward secrecy*: the proposed scheme computes the session key between the user_*i*_ and server_*j*_ as follows:
  (16) $$\begin{array}{c} A_{i}=D_{i}⊕\text{PSK}⊕h\left(\text{PSK}\right),\\ N_{1}=M_{1}⊕h^{2}\left(A_{i}\right)⊕h\left(\text{PSK}\right),\\ N_{2}=M_{3}⊕h^{2}\left(N_{1}\right)⊕h\left(\text{PSK}\right),\\ \text{S}{\text{K}}_{ij}=h\left(N_{1}||N_{2}\right). \end{array}$$
  Though the user's long-term key *A*_*i*_ is compromised, the adversary cannot compute *N*_1_ or *N*_2_ because the adversary cannot calculate the *h*(PSK) and PSK, so it cannot generate session key between user_*i*_ and server_*j*_. Therefore, the proposed scheme achieves perfect forward secrecy.  Table 1 shows the analysis on the security properties of various multisever authenticated key agreement schemes.

### 6.2. Attack Resistance

(A1) * Replay attack resistance:* the proposed scheme is secure against replay attack by adding the random nonce *N*_1_ and the timestamp *T*_*i*_ into the message. Though an adversary intercepts the previous authentication message 〈AUID_*i*_, *M*_1_, *M*_2_, *D*_*i*_, *T*_*i*_〉 and sends it to the server, the server can check the illegality of the request using checking *N*_1_ and *T*_*i*_ as follows:
  (17) $$\begin{array}{c} \text{checks}\;\;M_{2}=h\left(N_{1}||\text{AUI}{\text{D}}_{i}||D_{i}||\text{SI}{\text{D}}_{j}||T_{i}\right). \end{array}$$
  So the proposed scheme can prevent the replay attack using *N*_1_ and *T*_*i*_ because the adversary cannot compute another *M*_2_ in *T*_*i*_
(A2) * Modification attack resistance:* the adversary can intercept the authentication message and attempt to modify it for illegal authentication. Using a one-way hash function, the proposed scheme checks whether authentication information is modified or not. The adversary cannot obtain the random nonce *N*_*i*_ or *h*(PSK), so the adversary cannot compute a legitimate authentication message. Therefore, the server and user can check whether the authentication message is modified by the adversary or not. Therefore, the proposed scheme is secure against modification attack.
(A3) * Stolen-verifier attack resistanc:* the registration center and application servers do not have the user's ID/password table or the biometrics. The application server server_*j*_ authenticates the legitimate user using *h*(PSK) and *D*_*i*_. Therefore, the adversary cannot obtain the authentication information about legitimate users even if the adversary gets the authority to access the database of the RC or application servers. Thus, proposed scheme is secure against stolen-verifier attack.
(A4) * Off-line guessing attack resistance:* an adversary can extract the information stored in smart card using a side-channel attack such as SPA or DPA. So the adversary can know UID_*i*_, *B*_*i*_, *C*_*i*_, *D*_*i*_, and *E*_*i*_, but he cannot figure out a user's password because *h*(PSK), PSK, BIO_*i*_, and *x* are unknown to the adversary. In proposed scheme, the user's password is always used with the biometrics of the user; *h*(PW_*i*_ ⊕ BIO_*i*_), which are protected by the one-way hash function. Therefore, the adversary cannot calculate the user's password because biometric information has high entropy. Moreover, the adversary cannot figure out the biometrics because it is impossible for any two people to have the same biometrics template. Therefore, the proposed scheme is secure on off-line guessing attack.
(A5) *Forgery attack resistance:* a legitimate user cannot attempt to forge another legitimate user. The legitimate user_*i*_ can know his parameters 〈UID_*i*_, *B*_*i*_, *C*_*i*_, *D*_i_, *E*_*i*_, PW_*i*_ and BIO_*i*_〉. However the user_*i*_ cannot calculate another user's real identity because another user's anonymous identity AUID_*i*_ changes in every session and is protected using a random nonce; AUID_*i*_ = *h*(*N*_1_) ⊕ UID_*i*_. Therefore, the proposed scheme is secure against the forgery attack.
(A6) * Insider attack resistance:* in the proposed scheme, the user_*i*_ never send plain PW_*i*_ and BIO_*i*_ to the registration center RC. The user_*i*_ sends only *h*(PW_*i*_ ⊕ BIO_*i*_), so the RC cannot obtain the user's password or biometrics. And the RC cannot compute the PW_*i*_ using *h*(PW_*i*_ ⊕ BIO_*i*_) because the biometric information has high entropy. Moreover, *h*(PW_*i*_ ⊕ BIO_*i*_) is sent through a secure channel and needs not store in the database of RC. So, it is difficult for even insider adversary to figure out user's PW_*i*_ and BIO_*i*_. Therefore, the proposed scheme is secure against the insider attack.
(A7) * Masquerade attack resistance: *the masquerade attack means that an adversary is authenticated with the legitimate server using a fake or real authentication information such as the authentication messages. In Chuang and Chen's scheme, the adversary uses the authentication message between user_*i*_ and server_*j*_ to gain unauthorized access of server_*k*_. This problem occurred because user_*i*_ and server_*j*_ cannot check the destination of authentication message. To solve this problem, the proposed scheme uses AUID_*i*_ and SID_*j*_ including *M*_2_ as follows:
  (18) $$\begin{array}{c} M_{2}=h\left(N_{1}||\text{AUI}{\text{D}}_{i}||D_{i}||\text{SI}{\text{D}}_{j}||T_{i}\right). \end{array}$$
  AUID_*i*_ includes UID_*i*_. So the server_*j*_ can check whether user_*i*_ wants to be authenticated with server_*j*_ or not. And also *M*_4_ include AUID_*i*_ and SID_*j*_ as follows:
  (19) $$\begin{array}{c} M_{4}=h\left(\text{SI}{\text{D}}_{j}||N_{2}||\text{AUI}{\text{D}}_{i}\right). \end{array}$$
  So the user_*i*_ can check whether server_*j*_ wants to be authenticated with user_*i*_ or not. The adversary cannot compute *M*_2_ and *M*_4_ because the adversary cannot compute *N*_1_ and *N*_2_. Therefore the proposed scheme is resistant to the masquerade attack.
(A8) * Smart card attack resistance:* in the proposed scheme, the smart card stores various information such as 〈UID_*i*_, *B*_*i*_, *C*_*i*_, *D*_*i*_, *E*_*i*_, *h*()〉. An adversary can obtain all information stored in user's smart card using SPA or DPA. But the adversary cannot compute the session key between user_*i*_ and server_*j*_ using *M*_1_ and *M*_3_ because the adversary cannot compute *h*(PSK) using obtained information as follows:
  (20) $$\begin{array}{c} N_{1}=M_{1}⊕h\left(B_{i}\right)⊕h\left(\text{PSK}\right),\\ N_{2}=M_{3}⊕h\left(N_{1}\right)⊕h\left(\text{PSK}\right),\\ \text{S}{\text{K}}_{ij}=h\left(N_{1}||N_{2}\right). \end{array}$$
  Though the adversary obtains *B*_*i*_ and *M*_1_, the adversary cannot compute *N*_1_ because of the ignorance about *h*(PSK). Thus the adversary cannot compute *N*_2_ and SK_*ij*_. Therefore the proposed scheme is secure against smart card attack.
(A9) * User impersonation attack resistance:* in Chuang and Chen's scheme, an adversary can impersonate the legitimate user using only user's smart card because the adversary can be authenticated to the server_*j*_ using user's smart card without user's password or biometrics. However, the proposed scheme uses *h*(PSK) for protecting *D*_*i*_, *N*_1_, *N*_2_, *M*_1_, and *M*_3_. For example, even though the adversary knows *M*_1_ and *B*_*i*_ in *M*_1_ = *N*_1_ ⊕ *h*(*B*_*i*_) ⊕ *h*(PSK), the adversary cannot compute *N*_1_ without *h*(PSK), so he cannot generate the SK_*ij*_. The adversary cannot know *h*(PSK) without user's password or biometric. So the adversary cannot impersonate a legal user. Therefore the proposed scheme is secure against the user impersonation attack.
(A10) * DoS attack resistance:* the proposed scheme checks the freshness of message using timestamp, so it is useless that an adversary sends the previous message to the server. Moreover, the proposed scheme uses *M*_2_ = *h*(*N*_1_||AUID_*i*_||*D*_*i*_||SID_*j*_||*T*_*i*_) that includes timestamp *T*_*i*_. The server can check the freshness and legality of *M*_2_ because *M*_2_ and the timestamp do not match even though the adversary sends the previous *M*_2_ with the current timestamp. Therefore the proposed scheme is more secure against the DoS attack than Chuang and Chen's scheme.

The proposed scheme is more secure than Chuang and Chen's scheme against the masquerade attack, smart card attack, user impersonation attack, and DoS attack, and also it achieves perfect forward secrecy. Moreover, the proposed scheme is also satisfactory with regard to the anonymity, mutual authentication, session key agreement, replay attack resistance, modification attack resistance, stolen-verifier attack resistance, off-line guessing attack resistance, forgery attack resistance, and insider attack resistance.  Table 2 shows the analysis on attack resistance of various multisever authenticated key agreement schemes.

### 6.3. Efficiency

The efficiency measures include single registration, simple and secure password modification, fast error detection, and low computational cost. In performance, the proposed scheme has similar computational with Chuang and Chen's scheme. Chuang and Chen's scheme has slightly lower computational cost than the proposed scheme, but it is vulnerable to various attacks. The proposed scheme has a little higher computational cost, but it is more secure than Chuang and Chen's scheme. In other words, the proposed scheme solves security problems using similar computational cost as compared with Chuang and Chen's scheme.

(E1) * Single registration:* in the proposed scheme, a user can be authenticated with various servers. However, the user does not need to register with every servers. To use the server's services, the user registers only one time with the registration center. The proposed scheme provides single registration so the user can anonymously use multiserver system using one registration.
(E2) * Simple and secure password modification:* in the proposed scheme, the user can change the user's password conveniently so that it is easy for the user to change the password anytime. And, the password change phase does not need any communication with the RC. Moreover, an adversary cannot change the password even though the adversary can obtain the smart card and the user's password. This is because that the smart card can check the incorrect biometric information using PW_*i*_, BIO_*i*_, *C*_*i*_, and *B*_*i*_. The smart card verifies whether *h*(PW_*i*_ ⊕ BIO_*i*_) ⊕ *C*_*i*_ is the same to *B*_*i*_ as follows:
  (21) $$\begin{array}{c} \text{checks}{\;\;B}_{i}=h\left(\text{P}{\text{W}}_{i}⊕\text{BI}{\text{O}}_{i}\right)⊕C_{i}. \end{array}$$
(E3) * Fast error detection:* during the login and password change phases, the smart card detects the error or mistake immediately when the adversary inputs the wrong identification, password, and biometrics information. The smart card can check the error or mistake without the RC's assistance. Therefore the proposed scheme provides fast error detection.

In Table 3, we use the following notations: “·”: that there is no computational cost in that phase, *n*: the number of users, *m*: the number of application servers, *C*_*h*_: executing time of one-way hash function, *C*_*F*_: executing time of the fuzzy extractor, *C*_ECC_: executing time of the elliptic curve encryption or decryption operation, and *C*_EXP_: executing time of the exponential operation. *C*_EXP_ is higher than *C*_ECC_. And *C*_EXP_ and *C*_ECC_ are considerably higher than *C*_*h*_. Therefore, the comparison of computational cost on above-mentioned operations is as follows:

(22) $$\begin{array}{c} C_{\text{EXP}}>C_{\text{ECC}}>C_{h}. \end{array}$$

And the hash function is generally executed quickly, so it is about 1000 times faster than asymmetric encryption. In D. Yang and B. Yang's scheme, the exponential operation is executed. In Yoon and Yoo's scheme, the elliptic curve encryption or decryption operation is executed. But in Chuang and Chen's scheme and proposed scheme, they use only one-way hash function. Therefore, Chuang and Chen's scheme and proposed scheme are faster than both D. Yang and B. Yang's scheme and Yoon and Yoo's scheme. And our proposed scheme adds only one *C*_*h*_ on RC's operation in the registration phase and also adds only one *C*_*h*_ on server's operation in authentication phase in comparison with Chuang and Chen's scheme. *C*_*h*_ has a little computational cost. Therefore, our proposed scheme has similar computational cost as compared with Chuang and Chen's scheme, but Chuang and Chen's scheme has security vulnerabilities on the masquerade attack, smart card attack, user impersonation attack, and DoS attack as well as no perfect forward secrecy. Our proposed scheme similarly maintains the computational performance and solves the security problems of Chuang and Chen's scheme. Therefore, the proposed scheme is the security enhanced anonymous multiserver authenticated key agreement scheme using the smart card and biometrics.

## 7. Conclusion

Chuang and Chen proposed an anonymous multiserver authenticated key agreement scheme. This scheme is efficient in that it only requires users to perform hash function evaluations but has various security vulnerabilities. So, we show that this scheme is vulnerable to a masquerade attack, a smart card attack, a user impersonation attack, and a DoS attack and does not achieve perfect forward secrecy. To solve the security problems of Chuang and Chen's scheme, we propose a security enhanced anonymous multiserver authenticated key agreement scheme using smart cards and biometrics. And also, we show how the security weaknesses of Chuang and Chen's scheme are addressed in our scheme and lastly analyze our scheme in terms of both security and efficiency.

## Figures and Tables

**Algorithm 1:**
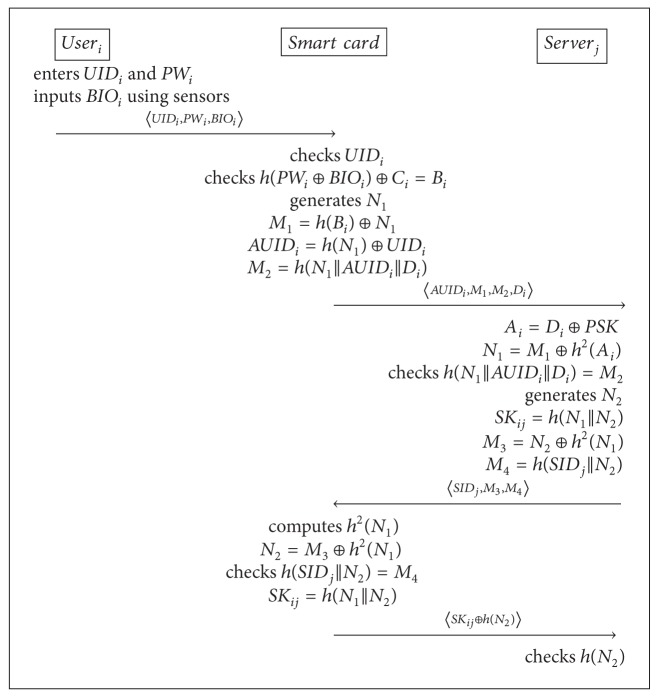
Login and authentication phase of Chuang and Chen's scheme.

**Algorithm 2:**
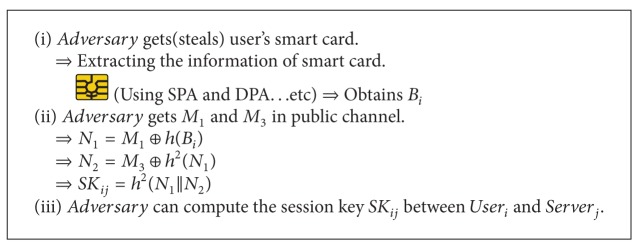
Smart card attack on Chuang and Chen's scheme.

**Algorithm 3:**
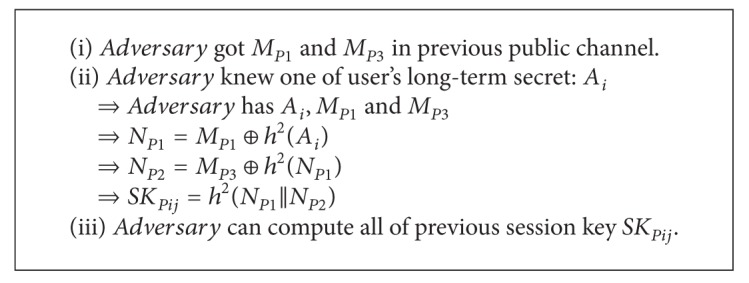
No perfect forward secrecy on Chuang and Chen's scheme.

**Algorithm 4:**
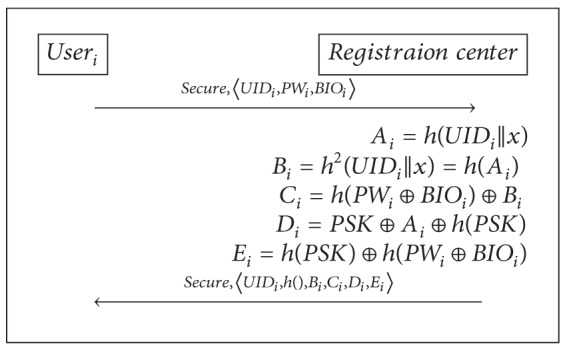
Our registration phase.

**Algorithm 5:**
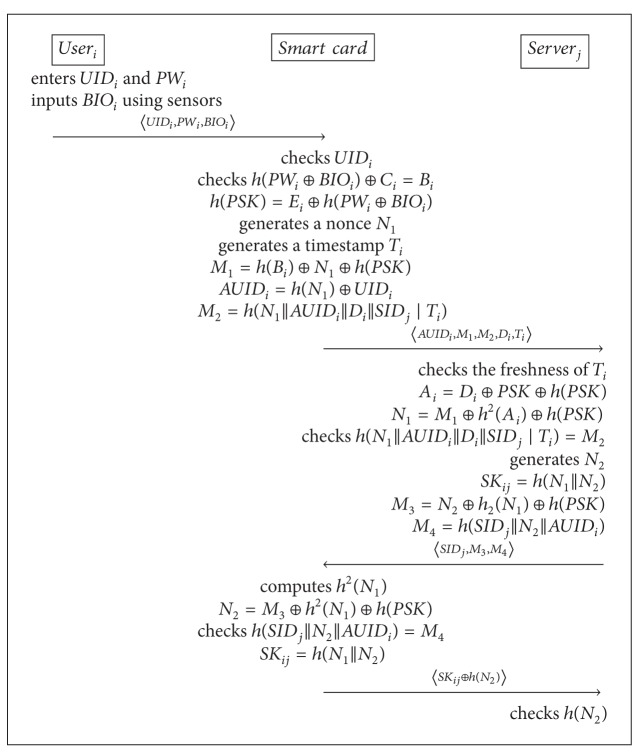
Our login and authentication phase.

**Algorithm 6:**
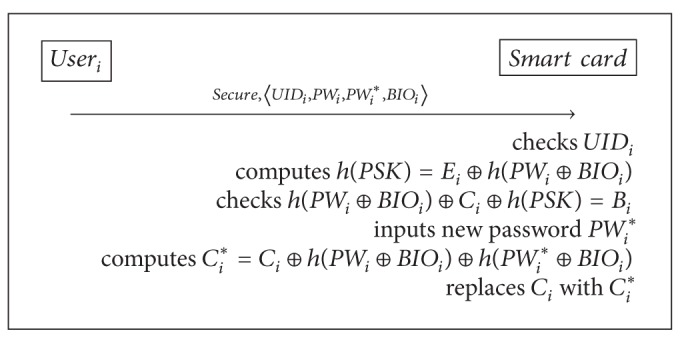
Our password change phase.

**Table 1:** Comparison of security properties.

Security properties	D. Yang and B. Yang scheme [10]	Yoon and Yoo scheme [11]	Chuang and Chen scheme [13]	Our scheme
(S1) Anonymity	×	×	◯	◯
(S2) Mutual authentication	◯	◯	◯	◯
(S3) Session key agreement	◯	◯	◯	◯
(S4) Perfect forward secrecy	◯	◯	◯	◯

**Table 2:** Comparison of attack resistance.

Attack resistance	D. Yang and B. Yang scheme [10]	Yoon and Yoo scheme [11]	Chuang and Chen scheme [13]	Our scheme
(A1) Replay attack	◯	×	◯	◯
(A2) Modification attack	◯	◯	◯	◯
(A3) Stolen-verifier attack	◯	◯	◯	◯
(A4) Off-line guessing attack	◯	×	◯	◯
(A5) Forgery attack	◯	×	◯	◯
(A6) Insider attack	×	×	◯	◯
(A7) Masquerade attack	×	×	×	◯
(A8) Smart card attack	◯	×	×	◯
(A9) User impersonation attack	◯	◯	×	◯
(A10) DoS attack	×	×	×	◯

**Table 3:** Comparison of efficiency measures.

Efficiency measures	D. Yang and B. Yang scheme [10]	Yoon and Yoo scheme [11]	Chuang and Chen scheme [13]	Our scheme
(E1) Single registration	◯	◯	◯	◯
(E2) S/S PW modification	◯	◯	◯	◯
(E3) Fast error detection	◯	◯	◯	◯

(E4) Low computational cost				
Registration user	·	*C*_*h*_	*C*_*h*_	*C*_*h*_
Registration server	·	·	·	·
Registration RC	*n*(3*C*_*h*_ + *C*_EXP_ + *C*_*F*_)	(*n* + *m*)*C*_*h*_	*n*(2*C*_*h*_)	*n*(2*C*_*h*_) + *C*_*h*_
Login user	4*C*_*h*_ + *C*_EXP_ + *C*_*F*_	2*C*_*h*_ + *C*_ECC_	4*C*_*h*_	4*C*_*h*_
Login server	·	·	·	·
Authentication user	*C*_*h*_ + *C*_EXP_	3*C*_*h*_ + *C*_ECC_	5*C*_*h*_	5*C*_*h*_
Authentication server	3*C*_*h*_ + 2*C*_EXP_	5*C*_*h*_ + 2*C*_ECC_	8*C*_*h*_	9*C*_*h*_
Authentication RC	·	7*C*_*h*_	·	·
PW change user	3*C*_*h*_ + *C*_*F*_	2*C*_*h*_	3*C*_*h*_	3*C*_*h*_
PW change RC	·	·	·	·

## Notations

*x*: A secret value of the registration center
RC: The registration center
UID_*i*_: The identification of user_*i*_
SID_*j*_: The identification of server_*j*_
AUID_*i*_: The anonymous identification of user_*i*_
PW_*i*_: The password of user_*i*_
BIO_*i*_: The biometrics information of user_*i*_
*h*(): A secure one-way hash function
*M*_*i*_: *i*th authenticator exchanged between user_*i*_ and server_*j*_
*N*_*i*_: A random nonce
PSK: A secure pre-shared key among RC and servers
||: A string concatenation operation
⊕: A string XOR operation
↔: Communication through a public channel
↔ Secure: Communication through a secure channel.
